# Association of phosphatase and tension homologue deleted on chromosome ten polymorphism rs1903858, but not serum levels with the risk of non–small‐cell lung cancer: A case‐control study

**DOI:** 10.1002/jcla.23328

**Published:** 2020-06-15

**Authors:** Zhen Liang, Yuzhu Tang, Hao Li, Youjun Xie, Lingling Zhan

**Affiliations:** ^1^ Department of Clinical Laboratory Red Cross Hospital of Yulin City Yulin China; ^2^ Department of Clinical Laboratory Ruikang Hospital Affiliated to Guangxi University of Chinese Medicine Nanning China; ^3^ Department of Blood Transfusion People's Hospital of Guangxi Zhuang Autonomous Region Nanning Guangxi China; ^4^ Department of Clinical Laboratory First Affiliated Hospital of Guangxi Medical University Nanning China

**Keywords:** gene polymorphism, non–small‐cell lung cancer (NSCLC), phosphatase and tension homologue deleted on chromosome ten (*PTEN*)

## Abstract

**Background:**

To investigate the association between phosphatase and tension homologue deleted on chromosome ten (*PTEN*) gene polymorphisms and non–small‐cell lung cancer (NSCLC) and further identify whether these polymorphisms influence serum PTEN levels.

**Methods:**

A total of 152 NSCLC patients and 124 healthy controls were included in the study. *PTEN* gene rs11202586 (T > C) and rs1903858 (A > G) polymorphisms were detected using the multiple single‐base extension technique (SNaPshot). The serum PTEN levels were determined using an enzyme‐linked immunosorbent assay (ELISA) kit.

**Results:**

The rs1903858 AG, GG genotypes, and G allele were associated with a higher risk of NSCLC (odds ratio (OR) =2.079, 95% confidence interval (CI) = 1.087‐3.974, *P* = .027; OR = 1.897, 95%CI = 1.053‐3.419, *P* = .033; OR = 1.505, 95%CI = 1.065‐2.126, *P* = .020). Stratified analysis reveal that the rs1903858 GG genotype and G allele were associated with an increased risk of squamous cell carcinoma (SCC) (OR = 3.226, 95%CI = 1.075‐9.678, *P* = .037; OR = 1.873, 95%CI = 1.092‐3.212, *P* = .023). Among smokers, the rs1903858 G allele carriers have an increased risk of NSCLC (OR = 1.916, 95%CI = 1.023‐3.589, *P* = .042), but a decreased risk of NSCLC was found with the AT haplotype. With respect to the serum PTEN levels, no significant difference was noted between NSCLC patients and healthy controls in this study.

**Conclusions:**

The study indicated that the rs1903858 gene polymorphism is associated with increased risk of NSCLC, particularly in SCC and smoker, and the haplotype AT was a protective factor for NSCLC. The serum PTEN levels were not associated with NSCLC.

## 1 INTRODUCTION

Lung cancer is the most common malignant; according to statistics in 2018, there were about 2.1 million new cases and 1.8 million deaths worldwide.^1^ Lung cancer has become the major cause of death in China, with the rising incidence and mortality.^2^

Lung cancer is triggered by a variety of factors. The major risk factors are environmental, cigarette smoking, and genetic. Polymorphisms in deoxyribonucleic acid (DNA) sequences caused by single‐base variations at the genome level are called single nucleotide polymorphisms (SNPs). The mutation of the SNP site may affect the function of the gene and lead to a change in its biological function and the occurrence of disease.^3^ As a tumor‐suppressor gene, phosphatase and tension homologue deleted on chromosome ten (*PTEN*) located on chromosome 10q23.3 and with a total length of 200 kb. Its encoded protein engages in the activities of both a protein phosphatase and a lipid phosphatase. *PTEN* is the tumor‐suppressor gene with bispecific phosphatase activity.^4^ Research suggests that *PTEN* has a variety of biological functions, for instance inhibiting angiogenesis, regulating cell proliferation, differentiation, and apoptosis. Thus, several studies have revealed a correlation between *PTEN* gene polymorphisms and various types of cancers, such as hepatocellular carcinoma, chronic myeloid leukemia (CML), and breast cancer.^5^, ^6^, ^7^ To date, three studies have found that *PTEN* gene polymorphisms were associated with the overall survival and sensitivity to chemoradiotherapy of NSCLC.^8^, ^9^, ^10^ Nevertheless, there are few studies concerning the linkage between *PTEN* SNPs and susceptibility to NSCLC.

Thus far, numerous studies have revealed that the change in *PTEN* activity is related to the occurrence, development, and metastasis of several cancers, such as gastric carcinoma, NSCLC, and breast carcinoma.^11^, ^12^, ^13^ Alimonti et al reported that the decrease in PTEN levels was associated with an increase in cancer susceptibility.^14^ A study by Wu et al suggested that the level of serum PTEN in acute myelocytic leukemia (AML) patients was closely related to the clinical stage and the degree of disease.^15^ However, there were no studies focused on the serum PTEN levels and the effect of *PTEN* polymorphisms on NSCLC.

The purpose of this study is to investigate whether SNPs rs11202586 and rs1903858 of the *PTEN* gene are associated with the risk of NSCLC, as well as to examine their influence on serum PTEN levels.

## 2 MATERIALS AND METHODS

### 2.1 Study population

A total of 152 NSCLC patients and 124 healthy controls were included in the study. The NSCLC patients were consecutively recruited between August 2018 and January 2019 from the First Affiliated Hospital of Guangxi Medical University (Guangxi, China). For the disease group, each patient was diagnosed with histologically confirmed NSCLC, including SCC and adenocarcinoma (AD), and excluded other concomitant tumors.^16^ The healthy control group matched the gender and age of the disease group, they did not have cancer or other serious illness, and healthy controls were recruited from healthy volunteers at the same hospital during the same time. Information was collected by questionnaire and electronic medical records. After interpretation of the study, all participants provided written informed consent. The study was approved by the ethics committee of the First Affiliated Hospital of Guangxi Medical University.

### 2.2 SNP selection

Single nucleotide polymorphisms genotype information was retrieved from the dbSNP data. The selected SNPs frequency were >0.05 and were previously described to be association with cancer.^17^

### 2.3 DNA extraction and polymorphism genotyping

About 2 mL of venous blood was collected from each participant and stored at −20°C before DNA extraction. DNA was isolated using the AxyPrep blood genomic DNA small dose kit (Axygen Biotech Co., Ltd.). DNA concentration and purity were determined by spectrophotometry and then stored at −80℃ before analysis. The multiple single‐base extension (SNaPshot) method was used to analyze the polymorphisms of the SNPs. The *PTEN* rs11202586 and rs1903858 polymorphisms were screened using a gradient thermocycler polymerase chain reaction (PCR) system. The primers were designed by the Primer 5.0 software. The PCR primers, single‐base extension primer sequences of sites of the *PTEN* gene, are listed in Table 1. The PCR was carried out in a total volume of 10 μL, including 0.1 μL of each specific primer (10 μmol\L), 2.0 μL Taq buffer (5×), 1.0 μL solution I (10×), 0.1 μL Hot Star polymerase (5U/μL), 1.0 μL Template (DNA), 0.8 μL deoxy‐ribonucleoside triphosphate (dNTP) (2.5 mmol\L), and 4.9 μL ddH_2_O. The conditions for PCR were described below: initial denaturation at 95°C for 15 minutes; 15 cycles of denaturation at 94°C for 40 seconds, annealing at 63°C (decreasing by 0.5°C per cycle) for 60 seconds, and extension at 72°C for 8 minutes; 25 cycles of denaturation at 94°C for 40 seconds, annealing at 56°C for 40 seconds, extension at 72°C for 90 seconds; and extension at 72°C for 8 minutes. The products were purified using the Shrimp Alkaline Phosphatase/exonuclease I (Exo I enzyme) method, and the final product was obtained using the SNaPshot Multiplex kit (ABI). This product was sequenced using ABI 3730, and the sequence was analyzed by Gene Marker V1.91 software, Liz120 being taken as the internal reference. Aim to determine the accuracy of the SNaPshot method, we randomly selected about 10% of the specimens for direct sequencing. The data analyzed by SNaPshot method were completely consistent with the direct sequencing results.

**TABLE 1:** PCR primers, single‐base extension primer sequences of sites of *PTEN* gene

SNPs	PCR primers	Single‐base extension primer sequences
rs11202586	F:5'‐TGTGTTATCTCATCTCTTATTCTTCC‐3'	Sep:5'‐GGGAAATACATTGTCCTAGAGTAGAA‐3'
	R: 5'‐TAACCCCCCAAAACAGACC‐3'	
rs1903858	F:5'‐GCAGCAATCAAACTAAAAGAATAC‐3'	Sep: 5'‐TACTCCAGCTATAGTGGGGAAA‐3'
	R: 5'‐TGTTTTGATTTTTGGTTTTTGAA‐3'	

Note

Abbreviations: F, forward; PCR, polymerase chain reaction; *PTEN*, phosphatase and tension homologue deleted on chromosome ten; R, reverse; SNPs, single nucleotide polymorphisms.

### 2.4 Measurement of serum PTEN levels

Serum samples from 113 patients with NSCLC and 94 controls were detected. About 3 mL of peripheral blood was obtained from each participant and collected into serum tubes. When the blood congealed, placed in a centrifuge and centrifuged at 3000 rpm for 10 minutes. The serum was collected into tubes and stored at −80°C before detect. The serum PTEN levels were detected by a double‐sandwich ELISA kit (Human PTEN ELISA KIT, Cat.#JL19145, Jianglai Biotech Company, Shanghai, China) following the manufacturer's instructions. The detection levels for PTEN range from 7.5 to 240 ng/mL, and the inter‐ and intra‐assay variation coefficients of the used kit in our study were 9% and 11%, respectively. To analyze serum PTEN levels, absorbance was read at 450 nm using an ELISA reader (680; Bio‐Rad).

### 2.5 Statistical analysis

All data were analyzed using statistical software SPSS version 23.0 (IBM Corp). A two‐sided *P* < .05 was accepted as statistically significant. Normally distributed variables were expressed as means ± standard deviations (SD), and the median and interquartile range (IQR) were used for the skewed variables. A Kruskal‐Wallis test was conducted to compare the differences among groups. The goodness‐of‐fit chi‐square test was used to estimate the Hardy‐Weinberg equilibrium (HWE). Genotype and allele frequencies of the two SNPs were compared between patients with NSCLC and controls by the chi‐square test (χ^2^). Binary logistic regression was used to calculate odds ratios (ORs) and 95% confidence intervals (CIs) after adjusting for gender, age, and smoking status, to assess the relative risk conferred by a particular allele and genotype.

## 3 RESULTS

### 3.1 Demographic and clinical characteristics of all the participants

A total of 152 NSCLC patients and 124 healthy controls were included in the study. The basic demographic and clinical characteristics of all the participants in the study are shown in Table 2. No significant difference was found between the two groups regarding gender and mean age. However, when considering the smoking status, the number of smokers in patients with NSCLC was higher than in healthy controls (*P* < .05).

**TABLE 2:** Demographic and clinical characteristics of the participants

Variables	NSCLC	Controls	*P* value
Total number	152	124	
Demographic parameters			
Age (mean ± SD)	57.43 ± 9.19	56.16 ± 9.05	.250
Gender			
Male	103 (67.8)	84 (67.7)	
Female	49 (32.2)	40 (32.3)	
Cigarette smoking			
Yes	62 (40.8)	31 (25.0)	.006
No	90 (59.2)	93 (75.0)	
Pathological types			
Squamous cell carcinoma	44 (28.9)		
Adenocarcinoma	108 (71.1)		

Note

Abbreviations: NSCLC, non–small‐cell lung cancer; SD, standard deviation.

### 3.2 *PTEN* rs11202586 and rs1903858 polymorphisms and the risk of NSCLC

The genotype distributions of the SNPs rs11202586 and rs1903858 in the *PTEN* gene were found to be consistent with the HWE in the controls (*P* > .05).

The genotype and allele frequencies of the *PTEN* rs11202586 and rs1903858 between NSCLC patients and healthy controls are presented in Table 3. Binary logistic regression analyses after adjusted for the gender, age, and smoking status showed that there was no association between the *PTEN* rs11202586 polymorphism and NSCLC risk in any analytic models. However, analysis for the *PTEN* rs1903858 polymorphism indicated that compared with the AA genotype, an increase risk of NSCLC was found for subjects carrying the AG and GG genotypes, with adjusted OR of 1.897 and 2.079, respectively. Similarly, a higher risk of NSCLC was found for subjects carrying the rs1903858 G allele (OR = 1.505, 95%CI = 1.065‐2.126, *P* = .020) than the A allele.

**TABLE 3:** Genotype distributions and allele frequencies of *PTEN* polymorphisms between case and controls

SNPs	NSCLC ( n = 152)	Control ( n = 124)	Adjusted OR （95%CI）	*P* value
rs11202586				
TT	66 (43.4)	64 (51.6)	1.0^ref^	
CT	71 (46.7)	50 (40.3)	1.293 (0.528‐3.166)	.575
CC	15 (9.9)	10 (8.1)	0.994 (0.402‐2.456)	.989
CT + CC	86 (56.6)	60 (48.4)	1.299 (0.797‐2.118)	.294
T allele	203 (66.8)	178 (71.8)	1.0^ref^	
C allele	101 (33.2)	70 (28.2)	1.194 (0.822‐1.735)	.353
rs1903858				
AA	37 (24.3)	39 (31.4)	1.0^ref^	
AG	57 (37.5)	55 (44.4)	2.079 (1.087‐3.974)	.027
GG	58 (38.2)	30 (24.2)	1.897 (1.053‐3.419)	.033
AG + GG	115 (75.7)	85 (68.6)	1.436 (0.833‐2.478)	.193
A allele	131 (43.1)	133 (53.6)	1.0^ref^	
G allele	173 (56.9)	115 (46.4)	1.505 (1.065‐2.126)	.020

Note

Abbreviations: CI, confidence interval; NSCLC, non–small‐cell lung cancer; OR, odds ratio; *PTEN*, phosphatase and tension homologue deleted on chromosome ten; SNPs, single nucleotide polymorphisms; χ^2^, chi‐square test.

### 3.3 Stratified analysis

The subjects were stratified according to pathological subtypes and smoking status to assess the effect of confounders on the association between the *PTEN* SNPs and the risk of NSCLC. Stratified by pathological subtypes reveal that compared with the AA genotype, subjects carrying rs1903858 GG genotype had an increased risk of SCC, with adjusted OR of 3.226 (95%CI = 1.075‐9.678, *P* = .037). In addition, the rs1903858 G allele carriers had a 1.873‐fold higher risk of SCC than the A allele. Nevertheless, no significant association was observed between the rs1903858 polymorphism and AD (*P* > .05). With regard to *PTEN* rs11202586 polymorphisms, the genotype and allele distributions among groups were not statistically significant. Stratification analyses of *PTEN* polymorphisms and risk of SCC and AD are listed in Table 4.

**TABLE 4:** Stratification analyses of *PTEN* polymorphisms and risk of SCC and AD

SNPs	SCC (n = 44)	AD (n = 108)	Control （n = 124）	SCC vs controls		AD vs controls	
				OR (95%CI)	*P* value	OR (95%CI)	*P* value
rs11202586							
TT	12	54	64	1.0^ref^		1.0^ref^	
CT	27	44	50	1.249( 0.177‐8.807)	.824	0.967 (0.231‐4.044)	.964
CC	5	10	10	0.322 (0.072‐1.439)	.138	0.858 (0.268‐2.745)	.796
CT + CC	32	54	60	2.724 (0.933‐7.951)	.067	0.837 (0.433‐1.620)	.598
T allele	51	152	178	1.0^ref^		1.0^ref^	
C allele	37	64	70	1.556 (0.901‐2.688)	.113	1.041 (0.692‐1.566)	.846
rs1903858							
AA	8	29	39	1.0^ref^		1.0^ref^	
AG	15	42	55	1.353 (0.251‐7.302)	.726	1.438 (0.426‐4.851)	.558
GG	21	37	30	3.226 (1.075‐9.678)	.037	1.787 (0.771‐4.143)	.176
AG + GG	36	79	85	0.670 (0.195‐2.304)	.525	1.141 (0.544‐2.392)	.727
A allele	31	100	133	1.0^ref^		1.0^ref^	
G allele	57	116	115	1.873 (1.092‐3.212)	.023	1.410 (0.971‐2.047)	.071

Note

Abbreviations: AD, adenocarcinoma; CI, confidence interval; NSCLC, non–small‐cell lung cancer; OR, odds ratio; *PTEN*, phosphatase and tension homologue deleted on chromosome ten; SCC, squamous cell carcinoma; SNP, single nucleotide polymorphisms.

Stratified analysis base on smoking status revealed that subjects have a history of smoking and with the rs1903858 G allele have a 1.916–fold higher risk of NSCLC than those carrying the A allele. However, the genotype distributions of rs11202586 in smoking subjects between NSCLC patients and controls were not statistically significant. Stratification analyses of *PTEN* polymorphisms and risk of NSCLC (smoker and nonsmoker) are shown in Table 5.

**TABLE 5:** Stratification analyses of *PTEN* polymorphisms and risk of NSCLC (Smoker and Nonsmoker)

SNPs	Smoker				Nonsmoker			
	NSCLC (n = 62)	Control (n = 31)	OR （95%CI）	*P* value	NSCLC (n = 90)	Control (n = 93)	OR （95%CI）	*P* value
rs11202586								
TT	26	14	1.0^ref^		40	50	1.0^ref^	
CT	29	13	0.788 (0.189‐3.286)	.743	42	37	1.673 (0.532‐5.261)	.379
CC	7	4	0.663 (0.159‐2.769)	.573	8	6	1.246 (0.391‐3.971)	.710
CT + CC	36	17	0.734 (0.227‐2.368)	.604	50	43	1.274 (0.608‐2.669)	.521
T allele	81	41	1.0^ref^		122	137	1.0^ref^	
C allele	36	21	0.975 (0.507‐1.875)	.939	58	49	1.298 (0.823‐2.047)	.262
rs1903858								
AA	13	10	1.0^ref^		24	29	1.0^ref^	
AG	22	14	2.984 (0.900‐9.889)	.074	35	41	1.732 (0.798‐3.760)	.165
GG	27	7	2.423 (0.828‐7.093)	.106	31	23	1.668 (0.818‐3.401)	.159
AG + GG	49	21	1.454 (0.404‐5.215)	.569	66	64	0.905 (0.396‐2.070)	.813
A allele	48	34	1.0^ref^		83	99	1.0^ref^	
G allele	76	28	1.916 (1.023‐3.589)	.042	97	87	1.376(0.908‐2.086)	.132

Note

Abbreviations: CI, confidence interval; NSCLC, non–small‐cell lung cancer; OR, odds ratio; *PTEN*, phosphatase and tension homologue deleted on chromosome ten; SNPs, single nucleotide polymorphisms.

### 3.4 Haplotype analysis

Haplotypes of the *PTEN* rs11202586 and rs1903858 polymorphisms were derived to detect haplotypes specifically correlated with NSCLC by using SHEsis software. The *PTEN* rs11202586 and rs1903858 haplotype frequencies (>3%) are listed in Table 6. Three haplotypes were observed, and the frequency of haplotype AT was associated with reduced NSCLC risk (OR = 0.661, 95%CI = 0.471‐0.928, *P* = .017). Haplotype AT may be protective for NSCLC.

**TABLE 6:** Analysis of *PTEN* haplotype frequencies with the risk of NSCLC

Haplotype	Case (frequency)	Control (frequency)	^2^	*P*	OR (95%CI)
AT	128(0.423)	129(0.521)	5.746	.017	0.661 (0.471‐0.928)
GC	98(0.324)	66(0.267)	1.965	.161	1.303 (0.900‐1.888)
GT	75(0.245)	49(0.197)	1.749	.186	1.317 (0.875‐1.981)

Note

Abbreviations: CI, confidence interval; NSCLC, non–small‐cell lung cancer; OR, odds ratio; *PTEN*, phosphatase and tension homologue deleted on chromosome ten.

### 3.5 Serum PTEN levels

As serum PTEN levels of the two groups were non‐normal distributed, they were presented as median ± IQR. The average PTEN levels in the NSCLC patients were 55.13 ± 69.98 ng/mL, and there were 42.3 ± 58.09 ng/mL in the healthy controls. No significant values was found when compared the serum PTEN levels between NSCLC patients and healthy controls (*P* > .05). When compared the serum PTEN levels among subjects with the same genotype in the two groups, no significant difference was observed. In addition, the difference of the serum PTEN levels among three different genotypes in the same group was not statistically significant. The data demonstrated that there was no significant association between serum PTEN levels and the rs1903858 and rs11202586 polymorphisms (Table 7).

**TABLE 7:** The association between *PTEN* gene polymorphisms and serum PTEN levels (median ± IQR, ng/mL)

Groups	Overall	rs1903858				rs11202586			
		AA	AG	GG	*P* values^a^	TT	TC	CC	*P* values^a^
NSCLC (113)	55.13 ± 69.98	53.97 ± 55.19	72.60 ± 79.30	51.91 ± 65.69	.049	54.97 ± 68.95	55.13 ± 71.00	57.59 ± 86.92	.881
Controls (94)	42.3 ± 58.09	34.08 ± 50.37	56.28 ± 48.71	37.89 ± 38.0	.093	41.09 ± 47.13	45.26 ± 55.54	29.96 ± 64.51	.422
*P* values^b^	0.058	0.079	0.223	0.275		0.090	0.636	0.261	

Note

Abbreviations: IQR, interquartile range; NSCLC, non–small‐cell lung cancer; *PTEN*, phosphatase and tension homologue deleted on chromosome ten.

^a^ Kruskal‐Wallis test, comparing the difference of serum PTEN levels among three different genotypes in the same group.

^b^ Mann‐Whitney *U* test, comparing the difference of serum PTEN levels in two groups among the subjects with the same genotype.

## 4 DISCUSSION

In recent years, although significant advances have been made in the diagnosis and treatment of NSCLC, due to the lack of typical symptoms in early‐stage NSCLC, the early diagnosis rate is only about 15%. Most patients have developed to the advanced stage, which is beyond the optimal treatment period, by the time the diagnosis is confirmed.^18^

Phosphatase and tension homologue deleted on chromosome ten has been shown to control the cell cycle and prevent excessive proliferation. Research has shown that *PTEN* functions mainly at the plasma membrane and the nucleus. At the membrane, *PTEN* has been known to regulate protein kinase B (*PKB*) activation and is dependent upon its phosphatase activity. In the nucleus, *PTEN* plays an important role in controlling DNA damage repair and maintaining chromatin enrichment independent of its phosphatase activity. As a tumor suppressor, mutation or defection in *PTEN* is one of the most important reason of malignant tumor, and these factors play an important part in the pathological processes of various types of malignant tumors.^19^, ^20^ A study by Liu et al predicted that *PTEN* polymorphism influence the expression of PTEN,^21^ and several researches found association between the expression of PTEN with lung and breast cancer.^22^, ^23^ This may be the underlying mechanism of the association between *PTEN* polymorphisms and multiple diseases. Recently, several studies have shown that *PTEN* gene polymorphism is related to the risk of various malignant tumors, such as liver cancer, CML, and breast cancer.^6^, ^7^, ^24^ However, there was few studies has been executed to research the relationship between *PTEN* polymorphisms and the serum PTEN levels and risk of NSCLC. Therefore, our study assesses this association.

Our data revealed a statistical significance relationship between *PTEN* rs1903858 polymorphisms and NSCLC risk. The presence of *PTEN* rs1903858 AG and GG genotypes was observed to significantly increase the risk of NSCLC. A similar association was found with the G allele. In addition, one haplotype AT was found to make a decreased risk of NSCLC. This finding showed that the haplotype AT may be protective against NSCLC. Although there have been no previous reports about the rs1903858 polymorphism and the risk of NSCLC, several SNPs in the *PTEN* gene have been demonstrated to be associated with NSCLC.^8^, ^9^, ^10^ A study by Hosgood et al suggested that rs1903858 polymorphisms were associated with a significantly reduction in the risk of chronic obstructive pulmonary disease, which is one of the risk factors for lung cancer.^25^ The difference may be due to the progression of the disease, the mutation of *PTEN* increased with tumor progression in patients. On the other hand, Hosgood et al only enrolled 53 cases, and the small sample size may explain this difference.

In a case‐control study, Andreassen et al provided evidence that the rs11202586 T allele may have an increased risk of a testicular germ cell tumor, with an OR of 1.16 (95%CI 1.06‐1.28; *P* = .04).^17^ However, there was no significant relationship found between the rs11202586 polymorphism and the risk of NSCLC in the study. The inconsistencies between our results and those of previous studies may be due to the different tumor types and the ethnicities of the study subjects.

Moreover, the data revealed the rs1903858 GG genotype and G allele have a significantly increased risk of SCC. But, there was no significant relationship has been found between the rs1903858 polymorphisms and the AD risk in the study. This result may be due to the different pathological tissues of lung cancer. According to the pathological types, lung cancer is mainly divided into small‐cell lung cancer, adenocarcinoma, squamous cell carcinoma, and large‐cell lung cancer.^26^ Jin et al revealed that the *PTEN* mutation rate was higher in squamous cell carcinoma than in adenocarcinoma (10.2% vs 1.7%, *P* = .02).^27^ Another explanation for this may be the limited number of NSCLC patients. After stratifying the subjects by pathological type, the sample size in each group was small.

As is well known, smoking is one of the most important environmental risk factors associated with the pathogenesis of lung cancer; a history of smoking is present in approximately 80% of lung cancer patients.^28^ In the current study, the subgroup analysis revealed a significantly increased risk of NSCLC among smokers with the *PTEN* rs1903858 G allele. This finding was in accordance with the idea mentioned in several previous studies.^27^, ^29^, ^30^

As far as we know, there have been no prior studies on the relationship between serum PTEN levels and NSCLC. Per our results, no statistical difference was detected when compared the serum PTEN levels between patients with NSCLC and healthy controls. When comparing the differences between serum PTEN levels and the two polymorphisms, no significant association was observed. Our data were neither consistent with previous studies which revealed a lower expression of PTEN in lung cancer tissues than the adjacent cancer tissues, nor are they in agreement with the findings on AML suggesting higher PTEN levels than healthy controls,^15^, ^31^ indicating that serum PTEN levels are a controversial result. Though the exact mechanism of this difference is not clear, previous studies have suggested that PTEN expression is regulated by many factors, such as ribonucleic acid (microRNA), Interleukin‐6 (IL‐6), and others.^32^, ^33^

The study has several potential limitations. First, the current study researched only two SNPs in the *PTEN* gene. Secondly, all subjects were from the same hospital and two ethnicities (Han and Zhuang) in Guangxi. Finally, the sample size in each subgroup was small when stratified analysis was performed. These limitations may influence the results of this study. Therefore, we need multi‐site and multi‐center samples to verify the experimental results.

In conclusion, our results indicate that the *PTEN* rs1903858 polymorphisms contribute to an increased risk of NSCLC, and the AT haplotype reduces the risk of NSCLC, which is a protective factor against NSCLC. However, there was no statistical difference of serum PTEN levels between patients with NSCLC and healthy controls, and no association between rs1903858 and rs11202586 polymorphisms and PTEN serum levels.

## CONFLICTS OF INTEREST

None.

## AUTHORS' CONTRIBUTION

The design and writing of this paper were completed by Zhen Liang. Yuzhu Tang and Hao Li performed the experiments. Youjun Xie analyzed the data. The manuscript was reviewed by Lingling Zhan.
